# Evaluation of the Biological Standardization of Native Der p 1, Der p 2 and Der p 23 Proteins Isolated from Natural Allergen Source

**DOI:** 10.3390/ijms27073332

**Published:** 2026-04-07

**Authors:** Ana I. Tabar, David Rodríguez, Evelyn Gutierrez-Suazo, E. Carolina Pinto, Cristina Pesántez-Méndez, Blanca E. Garcia, Paloma Martín, Gema Garcia, Ricardo Palacios, Montserrat Martínez-Gomariz

**Affiliations:** 1 Allergology Service, Universitary Hospital of Navarra, 31008 Pamplona, Spain; ana.tabar.purroy@navarra.es (A.I.T.); evelyn.gutierrez.suazo@navarra.es (E.G.-S.); ec.pinto.capote@navarra.es (E.C.P.); crissap_17@hotmail.com (C.P.-M.); blanca.garcia.figueroa@navarra.es (B.E.G.); 2 Institute for Health Research (IDISNA), 31008 Pamplona, Spain; 3 Research and Development Department at Diater Laboratorios, 28919 Madrid, Spain; d.rodriguez@diater.com (D.R.); p.martins@diater.com (P.M.); g.garcia@diater.com (G.G.); r.palacios@diater.com (R.P.)

**Keywords:** native allergen purification, molecular standardization, HDM

## Abstract

House dust mite allergens Der p 1, Der p 2, and Der p 23 are recognized as major clinically relevant allergens worldwide; however, it is difficult to obtain these proteins in purified form from a natural source, which limits their use in molecular targeted immunotherapy and in vivo diagnosis. In this study, we developed and validated robust methodologies for the large-scale purification and individual characterization of native nDer p 1, nDer p 2, and nDer p 23 allergens from the natural sensitization source, *Dermatophagoides pteronyssinus*. Each allergen was isolated through an independent downstream process based on successive chromatographic steps, achieving high purity and preserving the structural integrity. Molecular standardization was performed in vivo in 27 mite-allergic patients by skin prick testing (SPT), enabling the separate determination of histamine equivalent potency (HEP) values: 7.43 µg/mL for nDer p 1, 8.11 µg/mL for nDer p 2, and 1.55 µg/mL for nDer p 23. These data establish a direct relationship between the protein concentration and biological activity for each major allergen. In conclusion, the successful production and biological standardization of native nDer p 1, nDer p 2, and nDer p 23 proteins provide well-defined reagents for in vivo molecular diagnosis and enable more precise and reproducible standardization compared with complex allergen extracts.

## 1. Introduction

House dust mite (HDM) allergies affect over one hundred million individuals worldwide [1,2] and are recognized as one of the most important sources of indoor allergens causing allergic rhinitis and allergic asthma [3], particularly in children, with *Dermatophagoides pteronyssinus* and *Dermatophagoides farinae* accounting for the majority of clinically relevant HDM sensitization [4,5]. Allergen immunotherapy (AIT) is the only disease-modifying treatment that provides sustained efficacy for the symptoms of allergic patients [6]. AIT for patients with respiratory symptoms caused by house dust mite sensitization is currently based on the repeated administration of complex mite allergens derived either from whole mite cultures or isolated mite bodies. These whole-extract preparations contain mixtures of major and minor allergens, as well as non-allergenic components, and display substantial variability in composition and potency across manufacturers and batches [7,8]. The composition of the extracts in major allergens depends on the ratio of allergens in the allergenic source used, which all contribute to their biological potency [9]. Sometimes, to improve these mixtures, the sources are enriched with mite feces [10].

HDM allergens have been classified as homologous proteins from different mite species. Of the 40 allergens from *Dermatophagoides* spp. recognized by the International Union of Immunological Societies (IUIS) and the Allergen Nomenclature Subcommittee of the World Health Organization (WHO) (www.allergen.org, accessed on 7 January 2026), group 1, group 2, and group 23 allergens are considered major and clinically relevant allergens, contributing mainly to the allergenicity of the extract [11]. They are considered immunodominant, binding 50–70% of the amount of IgE to HDM extracts [12,13]. Evolution analysis of the *D. pteronyssinus*-specific IgE response has shown that these groups are among the earliest recognized allergens [12]. Der p 1 was the first identified dust mite allergen, purified by conventional chromatographic techniques [14]. It belongs to the cysteine proteases family, with a molecular weight of 24 kDa, and is present in high amounts in fecal pellets [15,16]. Der p 1 has high allergenicity, as shown by the specific IgE of 60–80% of patients allergic to HDM [13,17,18]. Early exposure to Der p 1 is significantly associated with the development of asthma during childhood [12,13]. Der p 2 shows an IgE antibody frequency of 70–90% [19,20] and is associated with allergic rhinitis [21]. This protein has structural and biochemical similarity to the MD-2 co-receptor of TLR4 and belongs to the NPC2 protein family [13,22], with a molecular weight of 14 kDa. Recently, the Der p 23 allergen, identified in the fecal envelope of mites and classified as a peritrophin [23], has been considered a major allergen. Der p 23 exhibits variable regional sensitization rates (42–84%) [18,19,20], yet it is strongly associated with asthma risk, particularly during childhood [12,24,25]. Furthermore, patients with persistent moderate-to-severe asthma exhibit a higher frequency of Der p 23 sensitization compared with those with milder forms of the disease [25].

The use of recombinant and native allergens for in vitro allergy diagnosis has made it possible to establish the seroprevalences of Der p 1, Der p 2, and Der p 23 allergens in mite-allergic populations, associating the sensitization profiles with clinical phenotypes [26,27] and the evolution of the pathologies [12]. Component-resolved diagnostics have demonstrated that most HDM-allergic patients are sensitized to combinations of Der p 1, Der p 2, and Der p 23 and that specific patterns (including Der p 23 monosensitization) may predict the asthma risk and AIT response [28,29].

It is difficult to isolate and purify these proteins on a pharmaceutical scale from the natural allergen source, which has limited their application as in vivo molecular diagnostic and molecular targeted immunotherapy, in contrast to other allergen sources such as the fungal allergen Alt a 1, which has successfully been developed as a molecular diagnostic and therapeutic marker [30]. This limitation has stimulated interest in the use of a purified native source, as an alternative to recombinant HDM allergens, to serve as a building block for next-generation molecular-defined vaccines, as well as for the development of more precisely standardized allergen products for diagnosis and immunotherapy [31].

Commercial *D. pteronyssinus* extracts display marked quantitative heterogeneity in the major allergens Der p 1 and Der p 2, with ELISA-measured concentrations spanning approximately 6.0–40.8 µg/mL for Der p 1 and 1.7–45.0 µg/mL for Der p 2 across manufacturers, despite their central role in defining HDM extract potency [7]. In contrast, the third serodominant fecal allergen Der p 23, now recognized as a major component with sensitization rates of 60–80% in many HDM-allergic populations [24,25], has still not been quantified in most diagnostic and therapeutic preparations [25,32]. Environmental measurements in household dust indicate that Der p 23 is present at lower absolute levels than Der p 1 and Der p 2 (≈1.9 vs. ≈7–8 µg/g) but strongly correlates with both allergens and mite counts, underscoring its contribution to the overall mite allergen burden [33].

Although the individual quantification of allergenic proteins provides valuable information for the characterization of whole extracts, their standardization is ultimately based on the biological potency, which reflects the combined contribution of all allergens present in the mixture, including both major and minor components. For this reason, variations in the concentrations of major allergens may still result in similar overall biological potency, thereby contributing to the observed heterogeneity between allergen extracts. This molecular and biological variability may impact both the diagnostic accuracy and the clinical efficacy and safety of AIT, highlighting the need for refined approaches for extract characterization and standardization [34].

Biological standardization using skin prick testing offers a functional integrative measure of the extract potency when performed according to the Nordic guidelines. It links the administered allergen dose to the in vivo potency in a clinically meaningful and reproducible manner [35]. In this approach, SPT is carried out using serial dilutions of the native purified allergen preparation tested in allergic patients alongside a fixed histamine control, and the dose–response relationship is quantitatively analyzed. The native purified allergen concentration that induced a wheal equivalent to that produced by histamine 10 mg/mL was determined. This parameter reflects the biological activity per protein concentration, enabling comparison between preparations and ensuring the batch-to-batch consistency of allergen extracts.

This study describes the methodology developed for the large-scale purification of nDer p 1, nDer p 2, and nDer p 23 allergens from the natural source of sensitization, as well as their individual characterization. Molecular standardization has allowed the independent determination of the biological potency of each protein and the establishment of standardized concentration values for each allergen.

## 2. Results

The purification processes for the nDer p 1, nDer p 2, and nDer p 23 allergens were carried out using three independent purification processes consisting of successive chromatographies that enriched the sample of the target protein.

**Figure 1:**
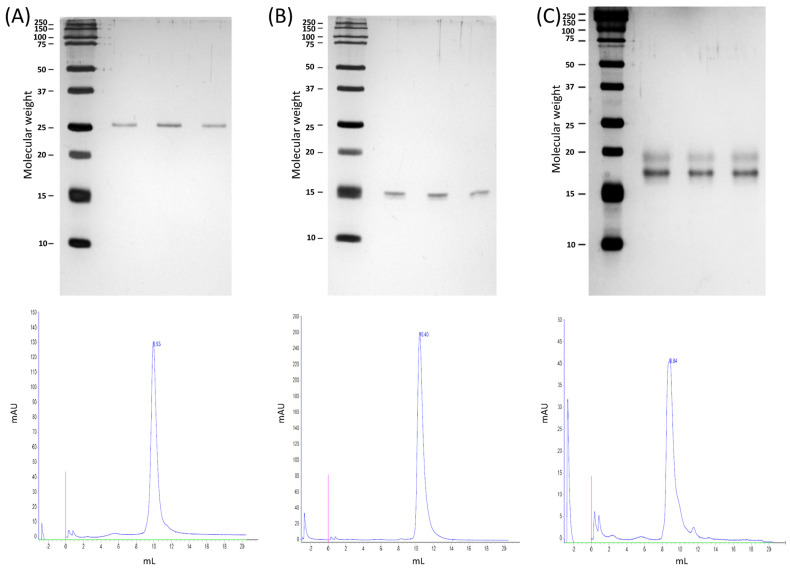
SDS-PAGE showing three different batches (top line) and representative analytical SEC chromatograms (bottom line) for the purification products of the chromatographic sequences proposed for nDer p 1 (**A**), nDer p 2 (**B**), and nDer p 23 (**C**).

nDer p 1 and nDer p 23 were obtained from complete mite cultures, while nDer p 2 was obtained from mite bodies isolated from the culture medium. nDer p 2 and nDer p 23 were purified using hydrophobic interaction chromatography, anion exchange chromatography, and a final purification step with molecular exclusion chromatography (SEC). In the case of nDer p 1, the hydrosoluble extraction was further enriched via anion and cation-exchange chromatography, followed by molecular exclusion chromatography. All three chromatographic sequences provided reproducible results regarding the recovery rates and concentrations of the purified allergens. nDer p 1 and nDer p 2 showed a single-band protein profile, which corresponded to a chromatographic peak of more than 90% purity (Figure 1A,B). In the case of nDer p 23, more than one protein species was enriched with the proposed procedure, whose identification was confirmed via peptide mass fingerprinting as Der p 23 in both bands (Figure 1C, Table S1). Der p 1 and Der p 2 were also identified via mass spectrometry.

Twenty-seven patients with symptoms related to dust mite allergy and SPT positivity were recruited to study the biological activity of each purified allergen. The mean age of the population was 35 years, and 56% were women. The predominant clinical symptom was rhinoconjunctivitis, associated or not with asthma of different degrees (Table 1 and Table S2).

Molecular IgE data were not available for 2 of the 27 patients included, because serum samples were not collected for analysis. Among the remaining patients, the frequency of positive specific IgE (sIgE > 0.35 kUA/L) to the whole extract of *D. pteronyssinus* was 100%, where 76% were sensitized to Der p 1, 92% had sIgE to Der p 2 and 68% to Der p 23. Notably, the Der p 2-specific IgE levels showed the highest titers in the studied population (Table 2 and Table S2).

**Figure 2:**
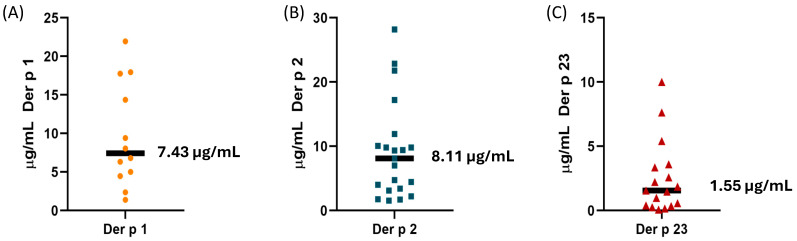
Distribution of HEPi values obtained in the standardization studies for nDer p1 (**A**), nDer p 2 (**B**), and nDer p 23 (**C**). The median HEPi value for each allergen (horizontal line) was calculated.

For the study of the biological standardization of each allergen, four protein concentrations were applied as one prick test per allergen. The wheal areas of 12 patients for nDer p 1, 21 patients for nDer p 2, and 17 patients for nDer p 23 were used to extrapolate the histamine wheal value [36]. The median of the individual HEP (HEPi) values was 7.43 µg/mL for nDer p 1, 8.11 µg/mL for nDer p 2, and 1.55 µg/mL for nDer p 23 (Figure 2).

## 3. Discussion

Historically, dust mite extracts have been obtained from complete cultures in which, through sieving processes, the larger fractions, containing whole mite bodies, have been separated from the smaller fractions, containing mainly feces. In this study, nDer p 1 and nDer p 23 were purified from complete cultures, while nDer p 2 was obtained from isolated mite bodies from the culture medium, in all cases with reproducible results regarding the recovery rates and concentrations of the purified allergens.

Biological standardization is relevant in the context of allergen extracts, where the proportion of major allergens can vary substantially [7,34]. According to the Nordic Guidelines [35], 27 patients with symptoms related to dust mite allergy were recruited to assess the biological activity of nDer p 1, nDer p 2, and nDer p 23 purified allergens. The guidelines recommend the inclusion of approximately 20–30 allergic patients in controlled biological standardization studies to ensure adequate methodological robustness and comparability. Nevertheless, the relatively limited sample size should be considered a potential limitation, as smaller cohorts may increase the risk of effect size overestimation and may limit the generalizability of the findings.

Most of the participants were adults (median 35 years) with a balanced sex distribution and predominantly presented with rhinoconjunctivitis. The sensitization profile observed in our cohort—76% for Der p 1, 92% for Der p 2, and 68% for Der p 23—is consistent with previous reports from European populations, confirming the immunodominance of Der p 2 and its relevance for both diagnosis and immunotherapy and the prevalence of the three allergens in the sensitized population [12,18,32,35].

The biological standardization of purified allergens using manual measuring wheals yielded a histamine equivalent concentration of 7.43 µg/mL for nDer p 1, 8.11 µg/mL for nDer p 2, and 1.55 µg/mL for nDer p 23, values that are directly related to their biological potency. These well-defined potencies contrast with the situation for commercial whole extracts of *D. pteronyssinus*, which exhibit substantial variability not only regarding their absolute amounts but also in their relative proportions, depending on the raw material and extraction conditions [7]. This heterogeneity restricts the accuracy of biological standardization based on whole extracts since the overall extract potency reflects the combined activity of all constituent allergens, including minor components, whose individual contributions differ markedly between products. Consequently, the HEP value of a whole extract cannot be directly extrapolated to the potency of each purified allergen, as it represents the integrated effect of the entire allergen composition [23].

Molecular in vitro diagnostics have transformed the field of allergology, offering significant advantages over conventional tests that rely on whole extract preparations [26]. This approach enables a more precise characterization of sensitization, particularly by differentiating between the primary sensitization to house dust mites and cross-reactivity. Our data indicate that this molecular paradigm can be extended from in vitro to in vivo diagnostics, specifically through skin prick testing using native purified dose-standardized allergens, which is supported by pediatric application data. In children sensitized to *D. pteronyssinus* with rhinitis, molecular profiling via SPT with nDer p 1, nDer p 2, and nDer p 23 serves as a valuable complement to the in vitro component-resolved diagnostics (CRD) [32]. This combination allows for the accurate definition of molecular sensitization profiles in the point of care and better aligns the diagnostic findings with immunotherapy selection.

Beyond diagnosis, the availability of biologically standardized purified allergens has important implications for allergen immunotherapy. The use of natural purified allergens for immunotherapy, once biologically standardized, represents a new milestone in specific molecular immunotherapy, as demonstrated in previous studies carried out with Alt a 1, the major allergen in *Alternaria alternata* [30], where a 63% decrease in the symptom and medication score was achieved. Furthermore, in patients who showed a positive clinical response, the specific IgE against the purified protein present in the immunotherapy, Alt a 1, decreased in almost all cases, regardless of whether the specific IgE to other allergens increased or decreased [37]. These findings support the concept that targeting single well-defined molecules can modulate the immune response in a highly specific manner.

In summary, we have established a robust industrial purification process for the allergens nDer p 1, nDer p 2, and nDer p 23, enabling the acquisition of these proteins in their native form and with the appropriate quality for clinical use, as an alternative to traditional whole extracts. This approach enables the determination of the individual potency of purified allergens according to in vivo biological standardization, directly correlating the allergen concentration with the biological activity. By focusing on single molecular components rather than complex whole extracts, our work provides a more precise and robust assessment of potency and offers a logical bridge between molecular diagnostics and molecular-defined immunotherapy. This strategy minimizes the effects of batch-to-batch variability in quantitative allergen composition. Together, these advances provide a solid foundation for the development of in vivo molecular diagnostic tests and set the stage for future clinical studies on the safety and efficacy of HDM-specific molecular immunotherapy.

## 4. Materials and Methods

### 4.1. Molecular Purified Allergen

The raw material for the purification procedures consisted of either complete cultures of *D. pteronyssinus* grown in animal protein-free culture media or purified mite bodies, all supplied by Allergen Servilab (Ávila, Spain).

For nDer p 1 purification, *D. pteronyssinus* culture extract, obtained via double protein extraction and diafiltration using Tris 20 mM, was applied on anionic exchange chromatography, using Hitrap QXL column (Cytiva, Marlborough, MA, USA). The nDer p 1 enriched fraction was loaded into a Hitrap SPFF column (Cytiva) to improve the purity, and the final fraction of nDer p 1 with a purity higher than 90% was obtained after size exclusion chromatography (Superdex 75 prep XK 50-60, Cytiva).

For nDer p 2 purification, the soluble fraction from *D. pteronyssinus* bodies resuspended in ammonium sulphate phosphate buffer was enriched in nDer p 2 with hydrophobic interaction chromatography (HIC), using a Hitrap Capto phenyl column (Cytiva). After a buffer exchange step (Sephadex G-25 resin column), a pool fraction containing Der p 2 was loaded for cation exchange chromatography (Hitrap SPFF column, Cytiva). The purity of the enriched fraction of nDer p 2 was finally refined using in-size exclusion chromatography (Superdex 75 prep XK 50-60, Cytiva) to obtain nDer p 2.

Regarding nDer p 23 purification, a double protein extraction sample from *D. pteronyssinus* culture was precipitated with ammonium sulphate 60% (*w*/*v*). After sample centrifugation, HIC was used as the first step to purify nDer p 23. The buffer was exchanged from the enriched fractions and conditioned in Gly 50 mM + NaCl 100 mM to perform cation exchange chromatography using a Hitrap SPFF column (Cytiva). Finally, the nDer p 23-enriched sample was conditioned via buffer exchange chromatography in Sephadex G-25 fine.

The allergen quantification of each allergen was measured using the specific ELISA 2.0 kit (Inbio, Cardiff, UK).

### 4.2. Identification of Proteins by MS

Proteins were identified via mass spectrometry at the UCM Proteomics facility. The resolved nDer p 1, nDer p 2, and nDer p 23 bands were in-gel tryptic digested [38]. Peptide mass fingerprints and MS/MS spectra were analyzed in a MALDI-TOF/TOF mass spectrometer 4800 plus Proteomics Analyzer (Applied Biosystems, MDS Sciex, Concord, ON, Canada) and searched against the UniProt *Dermatophagoides pteronyssinus* database using the Mascot search engine. Protein identifications were considered significant when the Mascot score exceeded the significance threshold (*p* < 0.05).

### 4.3. SDS-PAGE and Purity

The resulting purified allergens were separated via 15% SDS polyacrylamide gel electrophoresis (SDS-PAGE) under reducing conditions, according to Laemmli’s methods [39], and were visualized with silver staining (Pierce^TM^ Silver Stain kit, Thermo Fisher Scientific, Rockford, IL, USA) for protein profile determination.

The protein purity was determined via size exclusion chromatography, SEC, with a TSK G2000 SW HPLC column from Tosoh bioscience, Grove City, OH, USA. A 300 µL aliquot of each sample was collected and loaded into the HPLC column. The absorbance at 215 nm was used to track the protein elution. The area from the peaks detected during the HPLC run was integrated to determine the purity (UNICORN™ v 7.3 software), and the total peak area from nDer p 1, nDer p 2, or nDer p 23 divided by the total peak area corresponded to the purity.

### 4.4. Subjects

A total of 27 patients diagnosed with rhinitis and/or asthma due to hypersensitivity to mites, confirmed by positive SPT to *D. pteronyssinus* at the Hospital Universitario de Navarra, were recruited for the in vivo standardization of the nDer p 1, nDer p 2, and nDer p 23 purified allergens from *D. pteronyssinus*.

Patients were excluded if they had (i) received any form of immunotherapy within the previous 5 years; (ii) taken antihistamines or other medications known to interfere with skin testing within 7 days prior to testing; (iii) presented dermographism, active skin lesions, or other cutaneous conditions that could affect the interpretation of the skin test results; or (iv) had suspected or confirmed pregnancy.

Written informed consent was obtained before the skin testing and blood extraction from all the patients.

### 4.5. Evaluation of In Vivo Potency

The in vivo potency of the major purified allergen was determined by its allergenic activity in HEP value, following the Nordic guidelines [35,40], using the skin prick test to define the concentration of the purified allergen with the same potency as histamine hydrochloride (HCl) 10 mg/mL.

Four ten-fold concentrations of each allergen were tested in duplicate (direct and inverse) per patient in both arms. The dilutions were 50, 5, 0.5, and 0.05 µg/mL for nDer p 1; 80, 8, 0.8, and 0.08 µg/mL for nDer p 2; and 10, 1, 0.1, and 0.01 µg/mL for nDer p 23.

The concentration of the purified allergens resulting in a histamine-like wheal was calculated by linear regression on the logarithmic line of the allergen concentrations tested and the wheal area measurements at each concentration [36,40].

The median of the values of the tested patients corresponds to the HEP value of the allergen, which is equivalent to 10,000 BU/mL.

### 4.6. Determination of the Wheal Area

Fifteen minutes after each allergen application, the wheal responses were measured via planimetric measurement. The contour of each wheal was first outlined on the skin with a fine-tip marker and then transferred to paper, using adhesive tape to obtain a clear contrast for planimetric evaluation. The resulting paper templates were digitized, and the wheal area (mm^2^) was calculated using the measurement tools available in Adobe Acrobat Reader^®^ software.

### 4.7. Serological Analysis

The specific IgE against *D. pteronyssinus* (d1), Der p 1 (d202), Der p 2 (d203), and Der p 23 (d209) was measured using ImmunoCAP (Phadia AB, Uppsala, Sweden) in individual serum from all patients included in the study. The levels ≥ 0.35 kUA/L were considered positive.

## Figures and Tables

**Table 1:** Description of the demographic characteristics and clinical diagnosis of the population recruited for the study (*n* = 27).

Age	Mean SD Min; max	35 12 18; 62
Sex	Male (*n*; %) Female (*n*; %)	12 (44%) 15 (56%)
Clinical Diagnosis	Rhinitis (*n*; %) Rhinoconjunctivitis (*n*; %) Rhinoconjunctivitis and mild asthma (*n*; %) Rhinoconjunctivitis and moderate asthma (*n*; %)	3 (11%) 16 (59%) 5 (19%) 3 (11%)

**Table 2:** Results of the determination of allergen-specific IgE in the study population (*n* = 25/27).

Allergen	Mean (kUA/L)	Median (kUA/L)	Max–Min (kUA/L)	Number of Patients with >0.35 kUA/L	Frequency
*D. pteronyssinus*	21.15	10.00	100–0.59	25	100%
Der p 1	11.08	2.81	82–0.00	19	76%
Der p 2	15.08	7.79	65.8–0.05	23	92%
Der p 23	2.7	1.03	23.2–0.00	17	68%

## Data Availability

The original contributions presented in this study are included in the article/Supplementary Materials. Further inquiries can be directed to the corresponding author.

## Supplementary Materials

The following supporting information can be downloaded at: https://www.mdpi.com/article/10.3390/ijms27073332/s1.

## Abbreviations

The following abbreviations are used in this manuscript:

AIT	Allergen immunotherapy
CRD	Component-resolved diagnostics
HDM	House dust mite
HEP	Histamine equivalent skin prick test
HEPi	Individual HEP
MS	Mass spectrometry
SDS-PAGE	Sodium dodecyl sulfate–polyacrylamide gel electrophoresis
SPT	Skin prick test
