# Supplementary material for: Evaluation of the Biological Standardization of Native Der p 1, Der p 2 and Der p 23 Proteins Isolated from Natural Allergen Source
Source: Int J Mol Sci. 2026 Apr 7;27(7):3332. doi: 10.3390/ijms27073332 (PMC13072800; doi:10.3390/ijms27073332)
Supplement: Supplementary file 1 [file ijms-27-03332-s001.zip › ijms-4174835-supplementary.pdf]

Supplementary Table S1: Protein identification by peptide fingerprinting using mass spectrometry. Protein bands/spots excised from SDS-PAGE gels (image below the table) were subjected to tryptic digestion and analyzed by MALDI-TOF MS. Proteins were identified using the Mascot search engine against the UniProt *Dermatophagoides pteronyssinus* database. Mascot score represents the probability-based score for protein identification. and the Expect value indicates the probability that the observed match occurred by chance. Sequence coverage represents the percentage of the protein sequence identified by matched peptides. Theoretical molecular weight (MW) and isoelectric point (pI) were obtained from UniProt entries.

Spot / Band	Protein name	Organism	UniProt Accession	Mascot Score	Expect value	Sequence coverage (%)	Matched peptides	Theoretical MW (kDa)	Theoretical pI
**1**	Der p 1 allergen (fragment)	*Dermatophagoides pteronyssinus*	A8DBQ9	83	6.3×10^{-5}	32	7	25.55	5.63
**2**	Der p 2 allergen (fragment)	*Dermatophagoides pteronyssinus*	Q3HWZ3	101	1.0×10^{-6}	73	9	14.42	7.10
**3**	Major mite allergen Der p 23	*Dermatophagoides pteronyssinus*	L7N6F8	68	0.0019	44	5	10.56	4.55
**4**	Major mite allergen Der p 23	*Dermatophagoides pteronyssinus*	L7N6F8	55	0.037	36	4	10.56	4.55

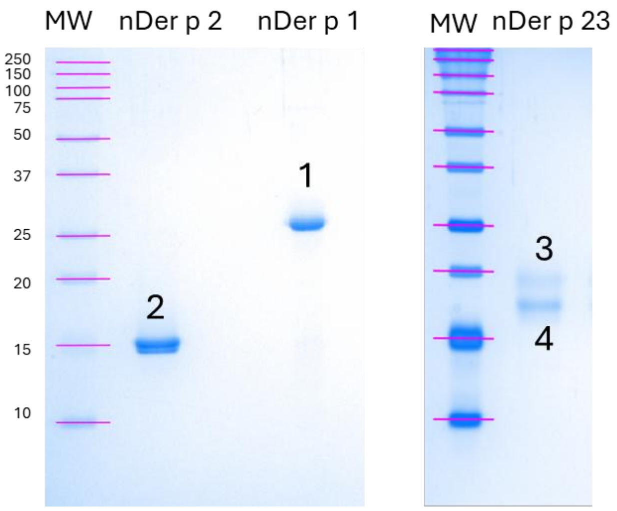

Supplementary Table S2: Demographic data. clinical diagnosis and specific IgE determination for each of the patients in the study. Specific IgE concentrations were determined using ImmunoCAP (Thermo Fisher Scientific) and are expressed in kUA/L. The positivity cut-off was 0.35 kUA/L. D. pteronyssinus: *Dermatophagoides pteronyssinus* extract. Der p 1. Der p 2 and Der p 23: molecular allergens from *D. pteronyssinus*. M: male; F: female.

				IgE (KUA/L)			
**Patient**	**Age**	**Sex**	**Clinical Diagnosis**	***D. pteronyssinus***	**Der p 1**	**Der p 2**	**Der p 23**
**101**	47	F	Rhinoconjunctivitis and mild Asthma	8.41	2.68	2.95	0.64
**102**	33	F	Rhinoconjunctivitis	21.6	2.98	14.4	0.99
**103**	27	F	Rhinoconjunctivitis	21.6	16.8	0.05	7.45
**104**	33	F	Rhinoconjunctivitis	13.7	2.06	4.62	0.02
**105**	20	F	Rhinoconjunctivitis	79.2	64.4	65.8	0.04
**106**	45	M	Rhinoconjunctivitis and mild Asthma	9.64	0.4	9.77	4.68
**107**	32	F	Rhinoconjunctivitis and moderate Asthma	10.1	2.02	7.15	3.06
**108**	23	F	Rhinoconjunctivitis and mild Asthma	0.59	0.01	0.2	0.36
**109**	23	M	Rhinoconjunctivitis	11.3	6.04	8.42	0.00
**110**	27	M	Rhinoconjunctivitis	4.54	0.02	6.68	0.00
**111**	57	F	Rhinoconjunctivitis	2.91	0.00	4.71	0.33
**112**	37	F	Rhinoconjunctivitis and mild Asthma	6.78	0.31	8.7	1.03
**113**	35	F	Rhinoconjunctivitis	>100	82	64.4	23.2
**114**	51	M	Rhinoconjunctivitis and moderate Asthma	9.03	0.17	4.76	1.57
**115**	53	M	Rhinoconjunctivitis and moderate Asthma	10	4.4	6.96	0.76
**116**	31	M	Rhinitis	60.7	20.6	50.5	5.18
**117**	25	F	Rhinoconjunctivitis	81	44.6	34.2	7.21
**118**	62	F	Rhinoconjunctivitis	5.04	1.61	3.88	0.26
**119**	18	M	Rhinoconjunctivitis	8.05	3.27	7.79	1.54
**120**	34	M	Rhinoconjunctivitis	11.1	3.05	8.96	1.85
**121**	20	F	Rhinitis	2.88	1.92	2.76	0.00
**122**	26	M	Rhinoconjunctivitis	NR	NR	NR	NR
**123**	30	M	Rhinoconjunctivitis	NR	NR	NR	NR
**124**	34	M	Rhinoconjunctivitis	17.9	4.32	18.8	2.35
**125**	52	F	Rhinitis	1.19	0.00	0.67	1.26
**126**	27	F	Rhinoconjunctivitis	21.6	2.81	31.1	3.61
**127**	39	M	Rhinoconjunctivitis and mild Asthma	9.94	10.6	8.65	0.01

NR: Not reported
